# Species delineation and genetic structure of two *Chaerephon* species (*C. pusillus* and *C. leucogaster*) on Madagascar and the Comoro archipelago

**DOI:** 10.1002/ece3.9566

**Published:** 2022-12-03

**Authors:** Morgane Tidière, Elodie Portanier, Stéphanie Jacquet, Steven M. Goodman, Gildas Monnier, Gregory Beuneux, Jean‐François Desmet, Cécile Kaerle, Guillaume Queney, Michel Barataud, Dominique Pontier

**Affiliations:** ^1^ LabEx ECOFECT Université de Lyon Lyon France; ^2^ LBBE UMR5558 CNRS Université de Lyon 1 Villeurbanne France; ^3^ Field Museum of Natural History Chicago Illinois USA; ^4^ Association Vahatra Antananarivo Madagascar; ^5^ Groupe Chiroptères Océan Indien La Saline France; ^6^ Société Française pour l'Etude et la Protection des Mammifères Bourges France; ^7^ Animal Genomics Laboratory ANTAGENE Lyon France; ^8^ Present address: Species360 Conservation Science Alliance Minneapolis Minnesota USA; ^9^ Present address: Interdisciplinary Centre on Population Dynamics University of Southern Denmark Odense M Denmark; ^10^ Present address: Department of Biology University of Southern Denmark Odense M Denmark; ^11^ Present address: CNRS, UMR 7144, Station Biologique de Roscoff Sorbonne Université Roscoff France

**Keywords:** cryptic species, cytochrome *b*, hybridization, microsatellites, Molossidae, population genetics

## Abstract

Cryptic species diversity is known to be common in bats but remains challenging to study in these mammals, whose natural history traits render their sampling and monitoring challenging. For these animals, indirect genetic approaches provide a powerful tool to gain insight into the evolutionary history and ecology of cryptic bat species. The speciation history of the polyphyletic *Chaerephon pumilus* species group (Molossidae) is poorly understood, including those found on western Indian Ocean islands. Two species in this complex have been identified in the Comoros: *C. pusillus* and *C. leucogaster*. Here, we aim to genetically characterize these two species and investigate their spatial population genetic structure. Analyzing five nuclear microsatellite markers from 200 individuals and one mitochondrial DNA gene (*Cyt‐b*) from 161 (out of the 200) individuals sampled on Madagascar and the Comoros, our findings indicated that these species are genetically differentiated. We observed mitonuclear discordance in numerous individuals (33% of the 161 _mt_DNA‐sequenced individuals). Based on ABC analyses, we found that this pattern could potentially be the result of asymmetric introgressive hybridization from *C. leucogaster* to *C. pusillus* and calls for further studies on the demographic history of these species. Moreover, at the intra‐specific level, analyses of the microsatellite loci suggested the evidence of a more pronounced, although weak, geographically based genetic structure in *C. pusillus* than in *C. leucogaster*. Altogether, our findings provide preliminary insights into the eco‐evolutionary aspects of this species complex and warrant further research to understand hybridization dynamics and mechanisms responsible for mitonuclear discordance.

## Graphical abstract

Cryptic species diversity is well‐known in bats but difficult to study: an indirect genetic approach provides a tool to understand their evolutionary and ecological history. Our study used both mitochondrial and nuclear genetic analyses to bring new insights into the evolutionary history of *Chaerephon leucogaster* and *Chaerephon pusillus* living on islands in the western Indian Ocean.

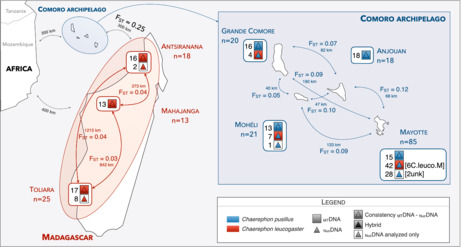

## 1 INTRODUCTION

Due to morphologically cryptic taxa (i.e., species that are genetically distinct but morphologically difficult to differentiate), biodiversity measures are often underestimated (Bickford et al., 2007). The direct study of the ecology of a given species or species complex can nevertheless be challenging, for example, when they have the overall capacity for daily movements, disperse over long distances, or are difficult to capture and recapture. DNA sequencing approaches provide insights into this hidden diversity (e.g., Hebert, Cywinska, et al., 2003; Hebert, Ratnasingham, et al., 2003). Once such cryptic species have been identified, studying aspects of their ecology and evolutionary history is of prime importance and provides knowledge for their management and conservation. The study of nonrecombining mitochondrial DNA does not always provide the correct determination of a species' phylogenetic relationships but does provide insights into the origin of currently observed populations and their colonization history (e.g., Ndiaye et al., 2016; Zahiri et al., 2019).

On the other hand, studying fast‐evolving genetic markers, such as microsatellites loci, is a powerful approach to inferring individual behavior (e.g., dispersal, sex‐biased dispersal, Moore et al., 2020), as well as the recent demographic history of populations (e.g., introductions or bottlenecks, Biebach & Keller, 2009; Portanier et al., 2017), and the interactions between the environment and gene flow (e.g., population and landscape genetics, Coulon et al., 2004; Portanier et al., 2018). Finally, since genetic diversity is linked to the adaptive potential of populations (Frankham et al., 2004) and thus to their persistence (e.g., inbreeding depression, Keller & Waller, 2002), the genetic characterization of populations helps to assess aspects related to their conservation needs. Such studies also help to determine whether hybridization occurs between different species, which is crucial when addressing species delimitations, especially in sympatric and cryptic species (e.g., Filippi‐Codaccioni et al., 2018).

In bats, much cryptic diversity is known to occur, which has been the focus of considerable research efforts in the last two decades (e.g., Ashrafi et al., 2013; Filippi‐Codaccioni et al., 2018; Jones, 1997). However, because of their nocturnal habits, generally small size and considerable mobility, bats are difficult to study and monitor in the wild. The genetic approach is thus an important method to provide a new understanding of these animals and has become extremely important in the field of conservation genetics (Dool, 2020; Tournayre et al., 2019). However, many bat genera and species complexes remain largely unstudied, especially for genetic markers (Dool, 2020).

**FIGURE 1:**
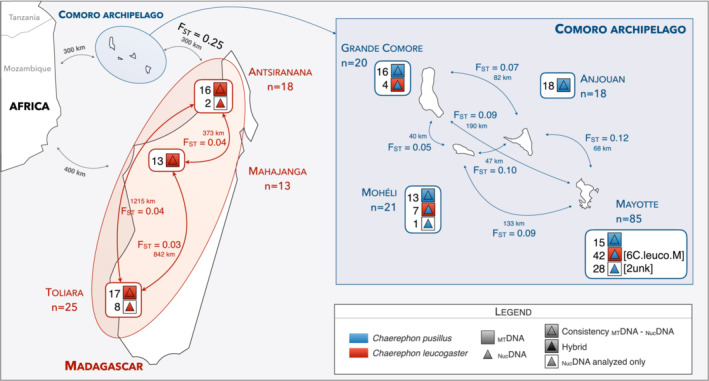
Summary of results based on the mitochondrial and nuclear markers of the 200 individuals of *Chaerephon* species analyzed from the Comoro archipelago and Madagascar. The number of individuals with different mitochondrial and nuclear DNA combinations is indicated for each site. C.leuco.M: individuals tentatively identified as *C. leucogaster* based on morphology. Unk: individuals not morphologically identified. Geographic (in km) and genetic (*F*_ST_) distances are indicated between and within species.

Among the chiropteran fauna on western Indian Ocean islands, the genus *Chaerephon* Dobson, 1874 (Molossidae) has a complex evolutionary history that is poorly understood. The *C. pumilus* species complex (Goodman, Buccas, et al., 2010) is a polyphyletic group (Goodman, Buccas, et al., 2010; Goodman & Ratrimomanarivo, 2007; Lamb et al., 2011; Taylor et al., 2009) showing high levels of phenotypic variation (Jacobs et al., 2004; Simmons, 2005). Two species belonging to this complex have been identified on islands in the Comoro archipelago: *C. pusillus* Miller, 1902, and *C. leucogaster* A. Grandidier, 1870. The distribution of *C. pusillus* is thought to be limited to the Comoros and western Seychelles (Aldabra Atoll and the Amirantes, Hutson, 2004; Lamb et al., 2011). By contrast, *C. leucogaster* is broadly distributed from Zanzibar, Pemba, and western Madagascar, and has recently been recorded in Mayotte (Maore) in the Comoros (Goodman & Cardiff, 2004; Ratrimomanarivo et al., 2009; Simmons, 2005; see Figure 1).

The Comoro archipelago comprises four islands (Grande Comore or Ngazidja, Mohéli or Mwali, Anjouan or Ndzuwani, and Mayotte, Figure 1) midway between Madagascar and the African continent, and separated by approximately 300 km of sea from these two areas. The Comoro islands were formed in situ by volcanic activity 15 million years ago (MYA; Emerick & Duncan, 1982, 1983): Mayotte is the oldest at 10–15 MYA, then Mohéli and Anjouan at 3.9–5 MYA, and Grande Comore is the youngest at 0.13–0.5 MYA (Emerick & Duncan, 1982; Nougier et al., 1986). The ring theory proposed by Naidoo et al. (2016) suggests that an African source population of the clade *C. pumilus*, for which genetic data suggest differentiated between 0.88 and 2.39 MYA, may have colonized the Comoro archipelago to form one arc of the ring and gave rise to *C. pusillus* species. The other arc of the ring probably colonized Madagascar and differentiated as *C. leucogaster* (among others, Goodman, Buccas, et al., 2010). The current sympatric distribution of *C. leucogaster* and *C. pusillus* (Goodman, Weyeneth, et al., 2010) in Mayotte raises the possibility of an expansion of *C. leucogaster* from Madagascar to the Comoros, thereby completing the ring.

Despite their complex evolutionary history, *Chaerephon* bats from the western Indian Ocean islands have only attracted research interest in the last two decades. Although the genus as currently defined is paraphyletic (Lamb et al., 2011), the *C. pumilus* group is monophyletic. The ecology and phylogeography for most species in this group remain poorly known, and species identification based on morphology is still provisional for specific taxa. Indeed, while cranial and dental characters have been used to distinguish *C. leucogaster* from *C. pusillus* (Goodman, Weyeneth, et al., 2010), the high degree of phenotypic variation observed in the genus (Jacobs et al., 2004; Simmons, 2005) makes species identification in some cases challenging, mainly where morphologically similar species occur in sympatry. This is the case in Mayotte, where both *C. leucogaster* and *C. pusillus* are present (Goodman, Weyeneth, et al., 2010). The presence of a white patch colouration in *C. leucogaster*, as compared to dark brown in *C. pusillus* and occasionally with a narrow white band, as well as differences in tragus shape, may be used to differentiate the two species in the field (Goodman, Weyeneth, et al., 2010; Ratrimomanarivo et al., 2009). Some individuals show mixed pelage characteristics, including white spotting in the brown fur, mainly on the ventrum, sometimes on the neck, the back, or the flank. This raises the question of hybridization between these species, which has not been tested using nuclear markers. Hence, genetic studies might provide new insights into the delimitation of these two species and additional information into the evolutionary history and ecology of the *C. pumilus* species group members. In the present study, we thus investigated the genetic variability and population structure of *C. leucogaster* and *C. pusillus* from Madagascar and the Comoros, using the mitochondrial cytochrome *b* gene (*Cyt‐b*, _mt_DNA) and five polymorphic nuclear microsatellite loci (_Nuc_DNA) for 161 and 200 individuals, respectively. Our research aimed to genetically characterize the cryptic species *C. leucogaster* and *C. pusillus* occurring on these islands, and describe their intra‐specific spatial population genetic structure.

## 2 MATERIALS AND METHODS

### 2.1 Sampling

Two hundred individuals of *Chaerephon* were sampled on islands of the Comoro archipelago (Grande Comore, Anjouan, Mohéli, and Mayotte, the first three islands part of the Union of the Comoros and the latter a French department), and in three areas of Madagascar (ex‐provinces of Antsiranana, Mahajanga, and Toliara; see Table 1 and Figure 1). Bats were captured using mist nets and harp traps. Captured individuals were removed from the trapping devices and placed in cloth bags until they were examined. According to current French legislation, the fieldwork in the Comoros was conducted by qualified people (trained ecologists, authorization n°2/09/DEAL/SEPR/2015 delivered by the Mayotte prefecture). Sex and morphometric measures (weight and forearm length) were recorded for each captured individual. Wing‐biopsy tissue samples were taken for all animals before being released at the capture site. For individuals captured in Mayotte, different pelage characters were noted (a photo taken with a digital camera for the 24 individuals that displayed a more or less extensive white spotting as described above, see Figure S1). The 200 sampled individuals included 116 females and 82 males. Six of these individuals captured in Mayotte were provisionally identified as *C. leucogaster* based on external morphology and referred to herein as “6 C.leuco.M” for “6 *C. leucogaster* from Mayotte” (Table 1). Finally, two individuals captured in Mayotte were not identified or sexed and were referred to as “2unk” (Table 1).

**TABLE 1:** The number of individuals of *Chaerephon* species analyzed and their morphological identification for populations from Madagascar (former province names listed) and the Comoro archipelago (see Figure 1).

Population	Island or ex‐province	N individuals	Morphological identification
Union of the Comoros	Grande Comore	20	59 *C. pusillus*
	Anjouan	18	
	Mohéli	21	
Mayotte		85	77 *C. pusillus*
			6 *C. leucogaster* from Mayotte (6 C.leuco.M)
			2 not identified (2unk)
Madagascar	Antsiranana	18	56 *C. leucogaster*
	Mahajanga	13	
	Toliara	25	

*Note*: A total of 116 females and 82 males were incorporated into this study. Two individuals have not been morphologically identified or sexed (2unk). In Mayotte, six individuals have been morphologically identified as *C. leucogaster* but with doubts (6 C.leuco.M).

### 2.2 DNA extraction and genotyping

DNA extraction of the 200 tissue samples was conducted according to the manufacturer's instructions (Nucleospin 96 Tissue Kit, Macherey‐Nagel). Briefly, sample tubes, including positive and negative extraction controls, were lysed overnight at 56°C, then DNA was isolated and purified using purification columns and vacuum filtration. DNA was eluted to obtain final concentrations between 20–100 ng/μl.

For each DNA sample, five microsatellites (TabrA10, TabrD10, TabrD15, TabrE9, and TabrH6) were amplified in simplex PCR using the primers indicated in Table S1 and analyzed with an automated sequencer. These five microsatellite markers were developed for a previous study on *C. pumilus* (Naidoo et al., 2016).

PCR amplifications were performed separately for each marker at 10 μl final volumes containing 5 μl of mastermix Taq Polymerase (Type‐It PCR Kit, Qiagen), 0.20 μM or 0.60 μM of the primers pair (depending on the marker, see Table S1) and a mean of 30 ng of genomic DNA. Each pair of primers was coupled with a fluorescent dye. Our PCR thermal protocol consisted of 95°C for 5 min, followed by seven touchdown cycles of 95°C for 30 s, 61°C to 55°C for 90 s (decreasing 1°C per cycle), and 72°C for 30 s, then followed by 29 cycles of 95°C for 30 s, 55°C for 90 s, and 72°C for 30 s, ending with an extension of 60°C for 30 min.

PCR products were resolved on a calibrated ABI PRISM 3130 XL capillary sequencer (ThermoFisher Scientific) under denaturing conditions (Hi‐DiTM Formamide; ThermoFisher Scientific) with an internal size marker prepared once and dispatched equally in all sample wells of each marker run. This internal size marker guarantees the same calibration for all samples. As the 200 samples were distributed on three plates and each plate contained the same positive reference controls (previously genotyped once), all positive controls were finally run four times on each marker, which guarantees both amplification and capillary resolution repeatability. The electropherograms were analyzed using GENEMAPPER 4.1 (ThermoFisher Scientific) and independently by two analysts to determine the allele sizes for each marker of each individual. When the genotypes determined by each analyst did not agree, the electropherograms were read again, and reading errors were resolved. In case of persistent disagreement, ambiguous results were considered as missing data. Genotype of each positive control was compared with its known reference to ensure the repeatability of analysis.

We also amplified the cytochrome *b* (*Cyt‐b*) mitochondrial gene (605 pb) for 161 individuals randomly chosen (46 individuals morphologically identified as *C. pusillus*, 109 as *C. leucogaster*, and 6 C.leuco.M; primers presented in Table S1). PCR amplifications were performed at 10 μl final volumes containing 5 μl of mastermix Taq Polymerase (Type‐It PCR Kit; Qiagen), 0.20 μM of the *Cyt‐b* nonfluorescent primers pair, and a mean of 30 ng of genomic DNA. The PCR thermal protocol consisted of 95°C for 5 min, followed by 40 cycles of 95°C for 30 s, 58°C for 90 s, and 72°C for 75 s, ending with an extension of 60°C for 30 min. Sequencing reactions were performed with each amplifying primers mentioned in Table S1 using a BigDye Terminator Cycle Sequencing Kit v.2.0 (ThermoFisher Scientific) following the manufacturer's protocol and running on an ABI 3130 XL capillary sequencer (ThermoFisher Scientific). The electropherograms were analyzed using Seqman Pro software (DNASTAR, Inc.).

### 2.3 Genetic analyses

#### 2.3.1 Genotyping errors and microsatellites characteristics

Genotyping errors (e.g., presence of null alleles, allelic loss) were tracked using the program MICROCHECKER v.2.2.3 (van Oosterhout et al., 2004). FSTAT v.2.9.3.2 (Goudet, 1995, 2001) was used to check for deviation from Hardy–Weinberg equilibrium for each locus (10,000 randomizations) and linkage disequilibrium for all pairs of loci (exact G‐tests). When necessary, p‐values were adjusted for multiple comparisons using the Bonferroni procedure (Bonferroni, 1936).

#### 2.3.2 Delimitation of species

**FIGURE 2:**
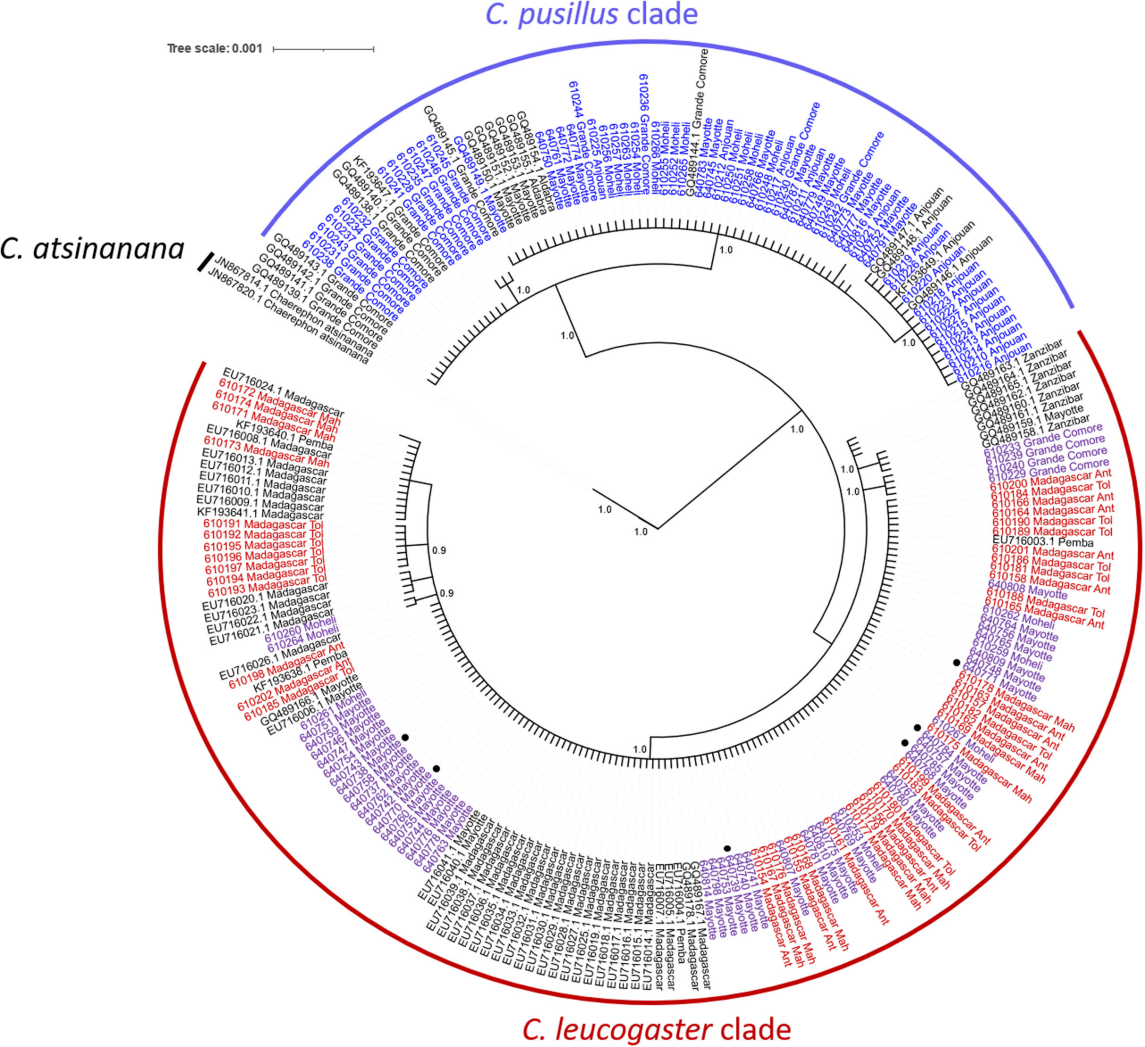
Bayesian phylogenetic tree of *Chaerephon* species based on cytochrome *b* sequences from 161 individuals sequenced herein and 75 sequences retrieved from Genbank. Node values correspond to posterior probabilities. Nodes supported by more than 0.9 are indicated. *Chaerephon atsinanana* is used as an outgroup. Individuals sequenced in this study are highlighted in blue and red for *C. pusillus* and *C. leucogaster*, respectively. The black‐colored sequences are publicly available sequences from both species. In purple, 53 individuals morphologically identified as *C. pusillus*, assigned to *C. pusillus* clusters based on _Nuc_DNA but carrying *C. leucogaster*_mt_DNA, referred to as *C. pusillus*‐*C. leucogaster* hybrids. From these 53 presumably hybrids bats, six (indicated with ●) individuals (6 C.leuco.M) were morphologically identified as *C. leucogaster*, while the remaining were morphologically assigned to *C. pusillus*. The Malagasy ex‐province locations are indicated as follows: Ant, Antsiranana; Tol, Toliara; and Mah, Mahajanga. The tree scale bar indicates the number of nucleotide substitutions per site.

To genetically verify the species identification, we first built a phylogenetic tree using the *Cyt‐b* sequences generated in this study and additional sequences retrieved from public databases (6 sequences of *C. pusillus*, 12 of *C. leucogaster*, and two sequences of *C. atsinanana* as an outgroup, Genbank database, NCBI, see Figure 2). The *Cyt‐b* sequences were aligned using the webPRANK server (http://www.ebi.ac.uk/goldman‐srv/webprank/) with default parameters (Löytynoja & Goldman, 2010). The phylogenetic tree was constructed using the Bayesian method implemented in MrBayes 3.2.7 software (Ronquist et al., 2012), with 10,000,000 generations. The automatic Smart Model Selection (SMS) in PhyML (Lefort et al., 2017) was employed to choose the adequate substitution model (HKY85 + G + I), based on Akaike Information Criterion (AIC, Burnham & Anderson, 2002). Since it has been proposed that *C. pumilus* has common ancestors that gave rise to *C. pusillus* and *C. leucogaster* species (Naidoo et al., 2016), we further constructed a phylogenetic tree including *Cyt‐b* sequences from *C. pumilus* to have an overview of the relationship between these regional species of *Chaerephon*. To decipher the genetic divergence between and within species, genetic distances (K2P) between pairs of *Cyt‐b* sequences were computed using ARLEQUIN v.3.5 software (Excoffier & Lischer, 2010).

Genetic clustering was also carried out using the five microsatellite markers mentioned earlier, developed by Naidoo et al. (2016). The Bayesian clustering algorithm implemented by the software STRUCTURE v.2.3.4 (Pritchard et al., 2000; Pritchard & Wen, 2010) was used to test the likelihood of different clustering solutions (*K*: number of clusters varying from one to 10, with 20 repetitions for each value of *K*). We used the admixture model with correlated allele frequencies and a burn‐in period of 300,000 steps followed by 1,000,000 MCMC repeats. The optimal number of genetic clusters was determined using both the log posterior probability of the data Ln Pr(*X*|*K*) and the Evanno ∆*K* method (Evanno et al., 2005) as implemented in the online software STRUCTURE HARVESTER v.0.6.94 (Earl & vonHoldt, 2012). Independent runs for the different *K* values were averaged using CLUMPP v.1.1.2 (Jakobsson & Rosenberg, 2007) as implemented in CLUMPAK (Kopelman et al., 2015), also implementing the DISTRUCT (Rosenberg, 2004) procedure to display graphical results. In CLUMPAK, the LargeKGreedy algorithm was used, with a random input order and 2000 repeats.

Genetic differentiation between the two species was measured using *F*_ST_ (theta estimator, Weir & Cockerham, 1984) calculated with FSTAT v.2.9.3.2 (Goudet, 1995, 2001). The significance level was assessed by exact *G*‐tests assuming random mating within samples with 10,000 permutations.

#### 2.3.3 Gene flow between species and historical demography

As potential incongruent patterns between mitonuclear markers could be the result of incomplete lineage sorting or hybridization, we tested whether gene flow between *C. pusillus* and *C. leucogaster* may explain the observed patterns (see Section 3) using an Approximate Bayesian Computation (ABC) framework (Beaumont et al., 2002). We ran the ABC analyses, with the ABCtoolbox package v.2.0 (Wegmann et al., 2010), and the programs SIMCOAL 2.0 and ARLSUMSTAT (Laval & Excoffier, 2004), on two different datasets. In the first dataset, the incongruent individuals were grouped with the *C. leucogaster* clade, whereas in the second set, they were included in the *C. pusillus* clade. All scenarios were run using the mtDNA (including *C. pumilus* sequences) and microsatellite loci. We tested three scenarios of species divergence: (i) an ancestral population splits into two distinct populations at time *t* with no genetic exchange between the descendant populations (Figure 4, model 1), (ii) an ancient asymmetric gene flow (at least 500,000 generations ago) between the descendent species (Figure 4, model 2), and (iii) recent asymmetric gene flow (less than 50,000 generations) between the two species (Figure 4, model 3). The demographic, historical, and mutation parameters drawn from the prior distributions are described in Table S2. A rejection sampling approach was used to simulate the data under each scenario. A set of summary statistics (for microsatellites: the mean number of alleles, mean total number of alleles, mean heterozygosity, mean total allelic range, pairwise *F*_ST_ and genetic distance (δμ)2; for *Cyt‐b*: standard deviation over loci of the heterozygosity for each population, mean heterozygosity, number of private polymorphic sites and pairwise *F*_ST_) were used to compare the simulated and observed data. To discriminate between the three models, we used a rejection method implemented in the R “*abc*” package (Csilléry et al., 2012).

We then evaluated the ABC performances in model choice and parameter estimates using the two following methods. First, we randomly picked 1000 pseudo‐observed datasets from all simulations generated for each scenario and determined how often the ABC procedure correctly predicted the correct scenario. Each pseudo‐observed dataset was used as the observed data to calculate the marginal densities of the different scenarios. Second, to evaluate how well the selected scenario fits with the observed data, we carried out a principal component analysis on the summary statistics computed from each model and plotted the observed data. Since the ring hypothesis postulates a population expansion of *C. leucogaster* from Madagascar to Mayotte, we examined the evolution of its population effective size using both the Bayesian skyline plot and Extended Bayesian skyline plot methods implemented in BEAST 2.5.0 (Drummond & Rambaut, 2007). The default priors and an HKY nucleotide substitution model were used. Chains were run for 500 million generations, the first 10% of which were discarded as burn‐in. We used TRACER 1.7.1 (Rambaut et al., 2018) for Bayesian skyline reconstructions with a 10% burn‐in.

#### 2.3.4 Within‐species genetic diversity

Microsatellite loci genetic diversity indices (*A*: the number of alleles per locus; AR: allelic richness, calculated using the rarefaction method; El Mousadik & Petit, 1996), observed (*H*_o_) and expected (*H*_e_) heterozygosities were estimated using the “*hierfstat*” package (Goudet & Jombart, 2001) in R v.4.0.1 (R Core Team, 2020). The within‐species population genetic structure was investigated using STRUCTURE with the same parameterization presented above. Species designation for each individual was based on the assignation resulting from the STRUCTURE method made at the inter‐species level. To test the importance of the different geographical zones of Madagascar (ex‐provinces) or islands of the Comoro archipelago in the structuration of *C. leucogaster* and *C. pusillus* populations, we computed global and pairwise *F*_ST_ values (theta estimator, Weir & Cockerham, 1984) between ex‐provinces (Madagascar) or Comoro archipelago using FSTAT v.2.9.3.2. (Goudet, 1995). Significance levels of global *F*_ST_ values were assessed by exact *G*‐tests assuming random mating within samples with 10,000 permutations. Pairwise *F*_ST_ values significance levels were evaluated using permutations, and *p*‐values were adjusted for multiple comparisons using the Bonferroni procedure (Bonferroni, 1936). Finally, isolation by distance inter‐specific patterns was assessed between geographic locations within species by regressing pairwise linearized *F*_ST_ (*F*_ST_/(1–*F*_ST_)) and log‐transformed geographic distances, using Mantel tests (9999 permutations, “*ecodist*” R package, Goslee & Urban, 2007), following Rousset (1997).

Genetic variability and population structuration were also determined from *Cyt‐b* sequences. First, intra‐specific diversity was evaluated by calculating nucleotide diversity (i.e., a set of genetic alleles usually transmitted together) per species with ARLEQUIN software. Subsequently, the haplotype network, allowing the reconstruction of genealogic links between haplotypes of each species, was built using the NETWORK software v.5.0.1.1, with the Median Joining method (fluxus‐engineering.com, Bandelt et al., 1999).

## 3 RESULTS

### 3.1 Microsatellite characterization

The five microsatellite markers were successfully amplified for the 200 bat samples with a mean amplification rate of 99.4%. All markers were polymorphic, with the number of alleles ranging between three and 35 (see Table 2). MICROCHECKER indicated low null allele frequencies (<0.10) for all loci in both species (Table S3). No allelic dropout was detected, but MICROCHECKER indicated that stuttering might have resulted in scoring errors in locus TabrA10 since very few heterozygotes were present (only two alleles were detected, separated by one base pair) in the *C. pusillus*. However, since the rarest allele was also found in *C. leucogaster*, leading to 26 heterozygous genotypes, we were confident that this allele was a real allele. No linkage disequilibrium between loci nor departures from HWE were detected in *C. pusillus* and *C. leucogaster* datasets. This was also indicated by low and nonsignificantly different from zero *F*_IS_ values (*F*_IS_ = 0.053, *p* = .04 and *F*_IS_ = 0.039, *p* = .17, respectively, adjusted nominal level = 0.01).

**TABLE 2:** Genetic diversity indices of the five microsatellite markers used in this study for two *Chaerephon* species.

Locus	*C. pusillus*				*C. leucogaster*			
	*A*	AR	*H*_e_	*H*_o_	*A*	AR	*H*_e_	*H*_o_
TabrA10	2	2.00	0.10	0.07	3	3.00	0.43	0.32
TabrD10	7	7.00	0.47	0.47	11	10.95	0.80	0.82
TabrD15	21	21.00	0.90	0.85	23	22.93	0.93	0.88
TabrE9	3	3.00	0.03	0.03	6	6.00	0.66	0.70
TabrH6	3	2.98	0.04	0.04	10	10.00	0.81	0.80
Mean ± SD	7.20 ± 7.95	7.19 ± 7.95	0.31 ± 0.38	0.30 ± 0.36	10.60 ± 7.64	10.58 ± 7.61	0.72 ± 0.19	0.70 ± 0.22

*Note*: The separation of the two species is based on morphological characteristics. The 6 C.leuco.M and 2unk individuals (see Table 1) were considered as *C. pusillus* here (see genetic clustering results).

Abbreviations: *A*, number of alleles per locus; AR, allelic richness; *H*_o_, observed heterozygosity; *H*_e_, expected heterozygosity.

### 3.2 Delimitation of species

Based on *Cyt‐b* polymorphism, phylogenetic reconstruction from the 179 *Chaerephon* (161 sampled individuals + 18 sequences from Genbank database) individuals suggested two highly supported clades (bootstrap 100%) corresponding to *C. leucogaster* and *C. pusillus* (Figure 2). These two clades showed a genetic divergence of 8.0% at the inter‐clade level but 2.0% and 1.3% within the *C. pusillus* and *C. leucogaster* clades, respectively. These divergence rates were similar when the introgressed individuals (see below) were excluded from the analysis, with an inter‐specific divergence of 7.42% and an intra‐specific divergence of 2.00% and 0.67% within *C. pusillus* and *C. leucogaster*, respectively. By including 59 sequences of *C. pumilus* from Genbank in the phylogenetic tree, we found the same distinct clades of *C. pusillus* and *C. leucogaster* (Figure S2).

**FIGURE 3:**
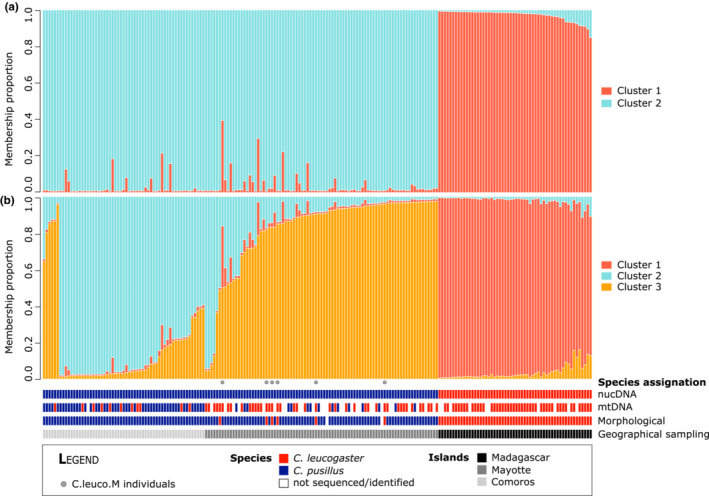
STRUCTURE clustering solution for (a) *K* = 2 and (b) *K* = 3 for the 200 individuals of *Chaerephon* species. Species assignment based on morphological, _mt_DNA, and _Nuc_DNA analyses is indicated for each individual and the island of capture. In (b), 81% of individuals in cluster 2 was captured in the Comoros, while 94% of individuals in cluster 3 was trapped in Mayotte.

Although our nuclear markers were limited to five polymorphic loci, the microsatellite data also supported this strong species distinction. STRUCTURE clustering procedure indicated an optimal number of clusters of *K* = 3 (maximum likelihood) or *K* = 2 (Evanno method, Figure S3A). When considering *K* = 2, the two clusters obtained coincide with the two species as morphologically identified (Figure 3a). Indeed, all of the individuals of *C. pusillus* clustered together (membership proportions ranging from 0.61 to 0.99, with only four individuals showing membership proportions lower than 0.8). Similarly, except for the 6 C.leuco.M individuals, all animals identified as *C. leucogaster* based on morphological characters clustered together (membership proportions ranging from 0.85 to 0.99). The 6 C.leuco.M and two undetermined individuals (2unk) were assigned to the *C. pusillus* cluster (cluster 1, Figure 3a) with high assignment probabilities (0.98–0.99 for the 6 C.leuco.M individuals and 0.99 for the 2unk when considering *K* = 2). When considering *K* = 3 (Figure 3b), all *C. leucogaster* (except the 6 C.leuco.M) were assigned to the same cluster, while *C. pusillus* were divided into two different clusters. One cluster (cluster 1) was mainly composed of animals captured on three of the islands in the Comoro archipelago (81%), specifically Grande Comore, Anjouan, and Mohéli. By contrast, the second cluster (cluster 3) was composed of individuals trapped in Mayotte (94%, including the 6 C.leuco.M individuals).

*F*_ST_ values were always high and significantly different from zero (exact *G*‐tests, *p* < .001, and IC95% not including zero, Table 3), suggesting that *C. pusillus* is genetically differentiated from *C. leucogaster*. The *F*_ST_ was slightly higher when the 6 C.leuco.M and 2unk individuals were included in *C. pusillus*. This follows the result from the STRUCTURE method, and the 6 C.leuco.M individuals were probably morphologically misidentified.

**TABLE 3:** Genetic differentiation between *Chaerephon leucogaster* and *C. pusillus* (based on both STRUCTURE assignment and morphological identification) with or without the 6 C.leuco.M and 2unk individuals (see main text) and considering these individuals in different species.

	*F*_ST_ value	95% CI	*p*‐Value
*C. leucogaster* (*n* = 56) vs. *C. pusillus* (*n* = 136)	0.261	0.077; 0.514	<.0001
*C. leucogaster* (*n* = 56) + 6 C.leuco.M vs. *C. pusillus* (*n* = 136)	0.211	0.064; 0.453	<.0001
*C. leucogaster* (*n* = 56) vs. *C. pusillus* (*n* = 136) + 6 C.leuco.M + 2unk	0.251	0.077; 0.524	<.0001

Given that private alleles are specific and unique to a population or species, they can be highly informative and allow the precise delimitation of species. We searched for private alleles based on our clustering results and identified two potential private alleles at locus TabrH6 in *C. leucogaster*.

While both markers discriminated between the two clusters, they nevertheless also displayed some incongruences between morphological and genetic species assignment. Among the 161 individuals sequenced for the *Cyt‐b* gene, 62 individuals were assigned to *C. pusillus* and 46 to *C. leucogaster* based on molecular (_mt_DNA and _Nuc_DNA) and morphological data (Figure 1). Interestingly, 53 of the remaining individuals (representing 33%) were differently assigned based on their mitochondrial and nuclear DNA: they carried _mt_DNA haplotypes of *C. leucogaster* while bearing *C. pusillus*_Nuc_DNA (stars, Figure 2). These bats represented 46% of the 115 individuals (62 pure *C. pusillus* + 53 _Nuc_DNA *C. pusillus*) bearing the _Nuc_DNA of *C. pusillus*. Among these 53 individuals, all sampled in the Comoros, 47 were morphologically identified as *C. pusillus* (Figure 2), and six were assigned to *C. leucogaster* (6 C.leuco.M, Figure 2).

Finally, the presence or absence of variously sized white spots or patches in the pelage of bats, most often on the ventrum, had no clear relationship with the genotype (6 C.leuco.M, four *C. pusillus*, and six hybrids between *C. leucogaster* and *C. pusillus*).

### 3.3 Gene flow between species and historical demography

**FIGURE 4:**
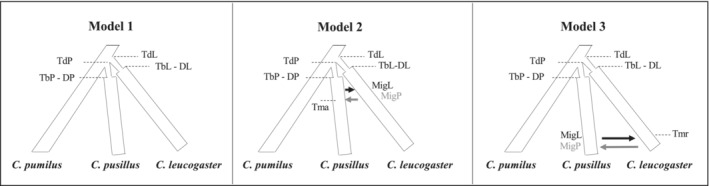
Graphical representation of the ABC models testing the demographic histories of *Chaerephon leucogaster* and *C. pusillus* divergence. TdP and TdL refer to the time of divergence events (in generation numbers, assuming a generation time of 5 years); Dbl and Dbp to the duration of bottleneck events; Tmr and Tma to the time of recent and ancient gene flow, respectively. Details on the parameter priors are given in Table S2.

Regardless of the dataset used for the ABC analysis, the posterior probabilities significantly selected model 3, which assumes recent gene flow between the species since their divergence (pp = 0.51–0.59; Figure 4). The two other models (Figure 4, models 1 and 2) had low posterior probabilities (pp = 0.14–0.15 and pp = 0.26–0.34, respectively) and were rejected. The goodness of fit between the selected model and the observed data was validated by the PCA analysis in which the simulated data fit well with the observed data (Figure S4). The ABC analyses showed a good power of discrimination between the three models (Figure S5), with a misclassification proportion of 0.35, 0.31, and 0.07 for models 1, 2, and 3, respectively. Most of the model confusions were found between models 1 and 3, which involved no gene flow and ancient migration. The time of secondary contact between the two species was estimated to be around 20,000 [1147;50,100] generations ago. The Bayesian skyline plot did not show any pattern of population expansion for either species (Figure S6).

### 3.4 Genetic species diversity

**FIGURE 5:**
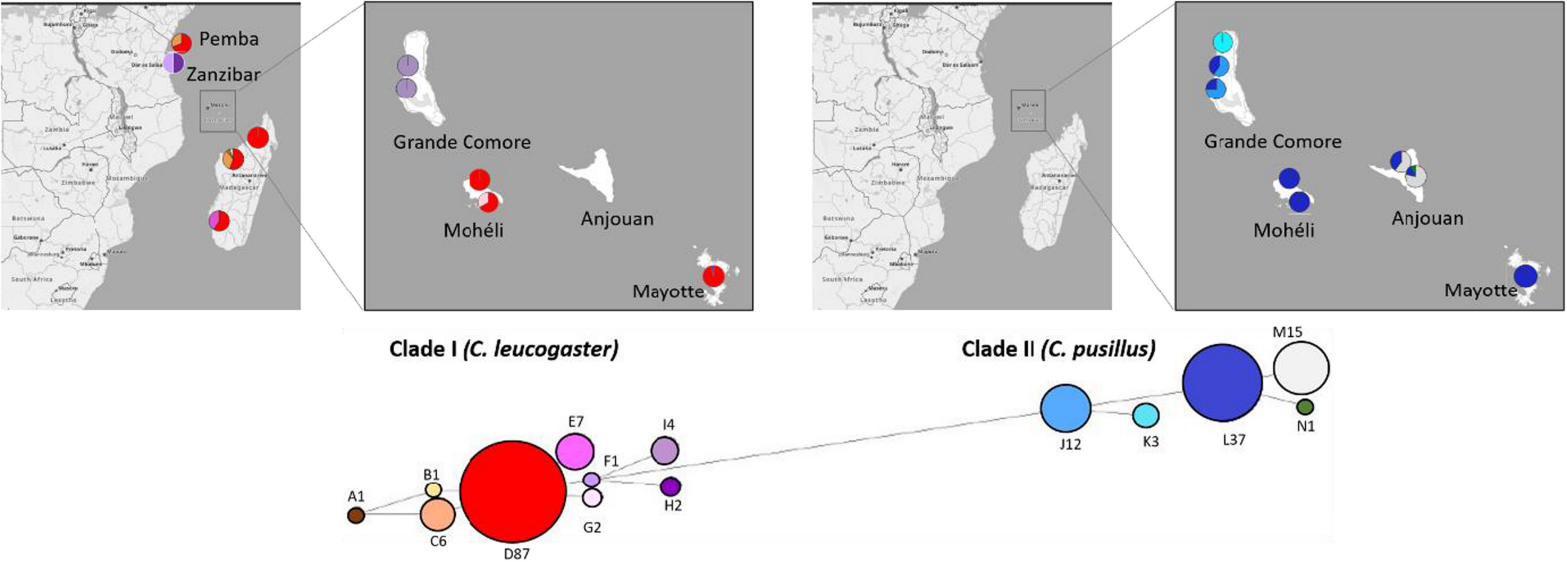
Haplotype network for the 161 individuals of *Chaerephon* species. Haplotype distribution of the two major clades corresponding to *C. leucogaster* and *C. pusillus* on Madagascar and in the Comoro archipelago. Letters correspond to the haplotypes, and numbers are the total individuals belonging to each haplotype.

We further investigated the genetic diversity and structure at the intra‐specific level for each species, assigning individuals to one species based on their assignment following the STRUCTURE analysis (i.e., the 6 C.Leuco.M were considered as *C. pusillus* and the 53 *C. pusillus* carrying *C. leucogaster*_mt_DNA were considered as *C. pusillus*). We found that the allelic richness of microsatellite loci tended to be lower in *C. pusillus* than in *C. leucogaster* (Table 2, Figure S7). Similarly, observed (*C. pusillus H*_0_ = 0.30 ± 0.36; *C. leucogaster H*_0_ = 0.70 ± 0.22) and expected (*C. pusillus H*_e_ = 0.31 ± 0.38; *C. leucogaster H*_e_ = 0.72 ± 0.19, Table 2) heterozygosities were considerably lower in *C. pusillus* than in *C. leucogaster*. Interestingly, this pattern was also observed with the *Cyt‐b* sequences for which nine haplotypes were identified in the *C. leucogaster* phylogenetic clade. At the same time, only five were detected in the *C. pusillus* clade (i.e., excluding the 47 individuals morphologically identified as *C. pusillus*, which carried *C. leucogaster* mitochondrial haplotypes, Figure 5). Nucleotide diversity in *C. leucogaster* populations was almost twice as high as in *C. pusillus* (*π* = 0.0022 and 0.0013, respectively).

Within‐species population structure was assessed through clustering analyses and *F*_ST_ computations. The optimal clustering solution assessed by a maximum likelihood was *K* = 1 for both *C. leucogaster* and *C. pusillus* populations, while Evanno's method indicated *K* = 2 and *K* = 3 for these species, respectively (Figures S8 and S9). When considering Evanno's clustering solutions, membership proportions in each cluster were around 50% (*K* = 2 for *C. leucogaster*) and 33% (*K* = 3 for *C. pusillus*; Figures S10 and S11), being consistent with the result obtained with the maximum likelihood method. Nevertheless, comparing the different sampling locations for each species (Madagascar ex‐provinces for *C. leucogaster* and the Comoro archipelago for *C. pusillus*), low but statistically significant global levels of differentiation (*F*_ST_ = 0.036 [0.017–0.051]_IC95%_ and *F*_ST_ = 0.096 [0.030–0.111]_IC95%_, respectively) were observed. For both species, all pairwise *F*_ST_ values were, in addition, significantly different from zero (Figure 1, Tables S4 and S5) and reached non‐negligible levels between the different island populations of *C. pusillus*. Genetic differentiation did not follow isolation by distance pattern (Mantel *r* = .22, *p*‐value = .75 for *C. pusillus*; Mantel *r* = −.74, *p*‐value = .67 for *C. leucogaster*).

Mitochondrial sequences also suggested a low level of within‐species genetic structure. While specific rare haplotypes were peculiar to some locations (e.g., M, N, and J for *C. pusillus* clade on Grande Comore and Anjouan; or F and G for *C. leucogaster* clade on Grande Comore and Mohéli), the most frequent haplotypes (L and D, present in 79% and 54% of *C. pusillus* and *C. leucogaster* individuals, respectively, Figure 5) were more widely distributed across the sampled area (Figure 5). Indeed, in *C. pusillus*, haplotype L was present across the Comoro archipelago (although not the most frequent on all islands). In Malagasy populations of *C. leucogaster*, haplotype D was predominantly found across the sampled area. Strikingly, three of the rare haplotypes (G, I, and H) in the *C. leucogaster* clade were found in individuals with *C. pusillus* nuclear DNA. These individuals showing mitonuclear discordance geographically overlapped with the *C. pusillus* haplotypes throughout the sampled area (Mohéli and Mayotte), except on Anjouan, from which none of the *C. leucogaster* haplotypes were found. This overlap of *C. leucogaster* and *C. pusillus* haplotypes in the Comoro archipelago was also evident by the co‐occurrence of the most frequent haplotypes of each clade in Mayotte.

## 4 DISCUSSION

Altogether, the genetic analyses performed in the present study indicated that *C. pusillus* and *C. leucogaster* from the Comoro archipelago and Madagascar are genetically distinct. Indeed, the mitochondrial phylogenetic reconstruction shows the existence of two strongly supported clades, mostly corresponding to individuals morphologically identified as *C. leucogaster* or *C. pusillus*, except 53 *C. pusillus* individuals showing evidence of introgressed _mt_DNA of *C. leucogaster*. This was further supported by a higher inter‐species divergence (7.4%) compared with the intra‐specific divergence (2.0% and 0.7% for *C*. *leucogaster* and *C*. *pusillus*, respectively). Likewise, this genetic distinction was also highlighted by microsatellite‐based population genetics analyses, which indicated the presence of two well‐supported genetic clusters corresponding to morphological identifications (except for the 6 C.leuco.M individuals). Accordingly, a solid genetic differentiation (*F*_ST_ = 0.25) was detected between the species, and, although this needs to be confirmed with a larger number of sample sizes and polymorphic markers, two alleles seemed private to *C. leucogaster*. Some inconsistencies were nevertheless detected, including (i) the assignment of individuals to one species based on morphological and _Nuc_DNA patterns, while carrying mitochondrial haplotypes of the other species, and (ii) the presence of individuals for which morphological identification and mitochondrial haplotypes were congruent but assigned to nuclear clusters of the other species (i.e., the 6 C.leuco.M individuals).

Morphological characteristics, for the most part, distinguish these species, and the scientific team of the “Société Française pour l'Etude et la Protection des Mammifères” (SFEPM) found that some individuals of *Chaerephon* in Mayotte can display white spots or patches in their pelage. In the present study, these individuals were genetically assigned to either *C. pusillus* or showed mitonuclear discordance between *C. leucogaster* and *C. pusillus*. Performing a Principal Components Analysis (PCA) based on four morphological characters (lengths of the forearm, the third and the fifth finger, and presence/absence of white pelage areas) systematically recorded in the field from 85 individuals captured in Mayotte, the results show no apparent difference between individuals with or without the white pelage pattern (see Figure S12). Furthermore, the six individuals identified in the field in Mayotte as *C. leucogaster* carried the mitochondrial *C. leucogaster* clade haplotypes but grouped with *C. pusillus* genetic cluster based on microsatellites. While species are traditionally described using morphological characters, these results highlight how genetic tools bring complementary information for species identification in this cryptic group.

### 4.1 Haplotype distribution of Cyt‐*b*

Genetics is also a helpful tool to unravel the colonization history of species. Regarding the complex *C. pumilus*, Naidoo et al. (2016) proposed the “ring hypothesis,” suggesting that *C. leucogaster* and *C. pusillus* evolved from a continental African ancestor of *C. pumilus*, which dispersed in two directions: (i) across the Mozambique Channel to the Comoro archipelago differentiating into *C. pusillus* and (ii) to Madagascar differentiating into *C. leucogaster*. While increasing the number of polymorphic loci will help to robustly infer the colonization history of *Chaerephon* species in the western Indian Ocean, the presence of *C. pusillus* carrying *C. leucogaster*_mt_DNA haplotypes in the Comoros (Figure 5) in the present study supports the ring hypothesis since the southernmost islands (Mayotte and Mohéli) harbored the same most frequent Madagascar haplotype (in red, Figure 5). By contrast, the population sampled in the northernmost island (Grande Comore) presents a haplotype found on the African continent and not on other islands that we sequenced material from. Although other explanations cannot be discarded (e.g., gene flow with different islands/African continent, larger effective population size), the lower diversity (in terms of the number of haplotypes) observed in the Comoros as compared to Madagascar might also suggest that colonization of the former is more recent than that of the latter (founder effect), further supporting the ring hypothesis. This source population could have migrated southward to Mohéli, Anjouan, and Mayotte, carrying only the L haplotype. Two additional haplotypes were nevertheless identified on Anjouan, which might have resulted from subsequent mutations or gene flow from nonsampled islands, such as Aldabra in the western Seychelles archipelago. However, the present study sample size and molecular markers were limited (i.e., a minimum of 18 individuals per island); therefore, increasing the number of samples and polymorphic markers is of utmost importance to test these hypotheses, and thoroughly examine the breadth of genetic diversity.

### 4.2 Mitonuclear discordance

Our present dataset suggests that 53 individuals out of 161 (33%) carried the *Cyt‐b* haplotype of *C. leucogaster* while bearing the _Nuc_DNA from *C. pusillus*. Interestingly, nearly half of the *C. pusillus* individuals sequenced for _mt_DNA bear the *Cyt‐b* haplotype of *C. leucogaster*, suggesting potential incomplete lineage sorting or asymmetric hybridization between species. While it is difficult to distinguish between the processes behind this pattern (e.g., Holder et al., 2001; Holland et al., 2008; Joly et al., 2009), our ABC analyses support a model involving recent gene flow rather than a scenario assuming strict reproductive isolation since their divergence. The fact that ambiguous individuals (i.e., 6 C.leuco.M) occur in specific geographical locations further suggests the hybridization model since incomplete lineage sorting would rather drive a random geographic distribution of these individuals.

Moreover, Naidoo et al. (2016) previously hypothesized that a population expansion of *C. leucogaster* from Madagascar to Mayotte led to secondary contact with *C. pusillus* in Mayotte, closing the colonization ring. Here, we found the largest proportion (74%) of individuals showing mitonuclear discordance in Mayotte compared with the other islands (20% and 35% on Grande Comore and Mohéli, respectively). Of note is that individuals displaying the lowest membership proportions using microsatellites and STRUCTURE software were also sampled in Mayotte (Figure 3). Notwithstanding, all the *C. leucogaster* sampled in Mayotte that showed ambiguous morphological traits (6 C.leuco.M individuals). These individuals carried the *C. leucogaster*_mt_DNA haplotypes, while grouping with the *C. pusillus* microsatellite‐based cluster, suggesting that they may be hybrids between the two species instead of *C. leucogaster* sensu stricto. No individual with a clear *C. leucogaster* phenotype was found in Mayotte, despite the sampling of numerous individuals during two expeditions: (i) in 2014 by the “Centre de recherche et de veille sur les maladies émergentes dans l'océan Indien” (CRVOI, Réunion Island, France; 77 individuals examined) and (ii) in 2015 by the SFEPM (480 individuals examined at 25 sites scattered across the island). More studies are required to ascertain the establishment of *C. leucogaster* in Mayotte and better characterize the hybridization pattern between these closely related species.

While hybridization is a broad phenomenon in bats due to their social life and mating system (e.g., Bogdanowicz et al., 2012; Mao et al., 2010), asymmetric hybridization seems to be less common with only a few reports, for example, between *Myotis myotis* and *M. blythii* (Berthier et al., 2006), or *Pteronotus rubiginosus* and *P. alitonus* (Filippi‐Codaccioni et al., 2018). Different processes may explain such a phenomenon, including disassortative mating, demographic dynamics of local and colonizing species, differential selection of mitochondrial variants, or a combination of factors (Toews & Brelsford, 2012). Deciphering the underlying mechanisms between *C. leucogaster* and *C. pusillus* requires further research, including behavior (dispersal bias, mating), population demography (difference in population size), and physiological features (genetic incompatibilities), especially within the zone of sympatry (Mayotte). Furthermore, a large‐scale analysis using markers from multiple genetic systems (e.g., mitochondrial, nuclear autosomes, and sex chromosomes), as well as environmental data, should provide the needed insights to disentangle the different ecological and evolutionary mechanisms that contribute to producing asymmetric introgression between these two species.

### 4.3 Within‐species genetic variability and population genetic structure

Within species, comparisons of genetic structure through clustering approaches suggested that individuals distributed across the sampled geographic areas of each species formed panmictic populations. However, it is noteworthy that although low, the *F*_ST_ values differed significantly from zero at the intra‐population level for both species (Figure 1, Tables S5 and S6). For *C. leucogaster* on Madagascar, the sampled area encompassed broad portions of the island, the most distant sampled sites being about 1200 km apart (between Antsiranana [Nosy Be] and Toliara). Keeping in mind the inference limitations due to the number of loci used in this study, the low or even absent genetic structure suggests that individuals of this species disperse over long distances, as highlighted by Ratrimomanarivo et al. (2009) based on mitochondrial *Cyt‐b*. Such weak or absence of genetic structure over broad geographical areas has also been reported in other bat species that are native to similar island systems (e.g., Carstens et al., 2002) and, notably, in western Indian Ocean bats such as flying fox species (*Pteropus s. comorensis* and *P. rufus*, Chan et al., 2011). Nevertheless, using D‐loop data, Ratrimomanarivo et al. (2009) found that *C*. *leucogaster* populations on Madagascar might be genetically structured because of climatically‐ and vegetational‐suitable regions separated by an unsuitable habitat that may impact dispersal and, hence, gene flow. This genetic structure could potentially mirror the *F*_ST_ significance we observed in our study. Although, these values and their level of significance could be biased by the low number of genetic markers and individuals involved in their analyses (e.g., Aguirre‐Liguori et al., 2020; Ruzzante, 1998). The genetic structure observed across Madagascar by Ratrimomanarivo et al. (2009) could also result, as suggested by the authors, in a sampling bias (i.e., unsampled areas between genetically differentiated populations). Further research using a higher number of nuclear markers and individuals is needed to confirm the observations of both studies and to determine whether such differentiation patterns are due to true gene flow barriers or isolation by distance and genetic drift‐gene flow equilibrium.

Significant *F*_ST_ values between islands in the Comoros were also detected for *C. pusillus*. Although the Mantel test did not support isolation by distance pattern, genetic differentiation in this species was higher between more distant islands, except for Anjouan. The latter was the most differentiated from the other islands in the archipelago, and genetic differentiation was not related to geographic distance (e.g., *F*_ST_ = 0.12 between Anjouan and Mayotte vs. *F*_ST_ = 0.05 between Grande Comore and Mohéli across similar spatial scales). This pattern was congruent with the results obtained with _mt_DNA: two specific mitochondrial haplotypes were found only on Anjouan, while Mohéli and Mayotte showed only one haplotype. These results suggest that the *C. pusillus* population on Anjouan is probably more isolated. A factor other than distance (e.g., wind direction, Wells, 2003) would facilitate the dispersal of individuals between the other Comoro islands. As mentioned earlier, another explanation could be that Anjouan might receive gene flow from other unsampled islands (e.g., Aldabra in Seychelles) or areas. The differences observed between *C. pusillus* and *C. leucogaster* might also suggest differences in dispersal behavior. Previous research on bat dispersal in island systems, including the western Indian Ocean islands, has detected species‐dependent patterns (e.g., Fleming et al., 2010; Moussy et al., 2013; Muscarella et al., 2011; Salgueiro et al., 2004; Speer et al., 2017). Some species are capable of long‐distance movements connecting populations across broad geographic distances (e.g., large bats such as *P. s. comorensis* and *P. rufus*, Chan et al., 2011), while open waters between islands (or between islands and the mainland) may pose a significant barrier to the dispersal of other species (e.g., small bats of the genus *Miniopterus* from Anjouan and Grande Comore, Weyeneth et al., 2008). As suggested for *C. leucogaster*, further studies with a larger sample size and a number of loci would be needed to confirm the pattern of genetic differentiation of *C. pusillus* between islands.

## 5 CONCLUSION

Our study brings new insights into the ecology and phylogeography of *C. leucogaster* and *C. pusillus* living on islands in the western Indian Ocean. The evolutionary history of these species from Madagascar and Comoros is complex. To advance even further on the aspects addressed in this paper, high‐throughput genome analyses will help uncover if contemporary hybridization occurs on the different islands (other than Mayotte), identify the classes of hybrids, and test alternative hypotheses that may explain the observed mitonuclear discordances. Indeed, for now, it is not possible to disentangle whether these discordances are the result of asymmetric hybridization or incomplete lineage sorting. Further studies using the approach of demographic inferences would allow discrimination between these processes and new insights into the population structure of *C. leucogaster* and *C. pusillus*. One example might include effective population size estimations, which might differ between species, as suggested by the observed differences in genetic diversity. Indeed, mitochondrial sequences and microsatellite loci consistently showed lower levels of genetic variation in *C. pusillus* than in *C. leucogaster*. Populations of *C. pusillus* inhabit small islands (from 290 km^2^ for Mohéli to 1659 km^2^ for Grande Comore) as compared to *C. leucogaster* occurring on Madagascar (587,041 km^2^), and smaller population sizes might thus be expected for *C. pusillus*, being more susceptible to genetic drift. Finally, our results indicate that some caution is needed for cryptic species, such as the two studied herein, regarding specific identification based only on pelage colouration and morphometric characters, further confounded by different measurement techniques employed by field workers, but these aspects are out of the scope of the current study.

## AUTHOR CONTRIBUTIONS

**Morgane Tidière:** Data curation (equal); formal analysis (equal); visualization (equal); writing – original draft (lead); writing – review and editing (lead). **Elodie Portanier:** Data curation (equal); formal analysis (equal); visualization (equal); writing – original draft (lead); writing – review and editing (equal). **Stéphanie Jacquet:** Data curation (equal); formal analysis (equal); visualization (equal); writing – original draft (equal); writing – review and editing (equal). **Steven M. Goodman:** Investigation (lead); writing – review and editing (supporting). **Gildas Monnier:** Investigation (supporting); writing – review and editing (supporting). **Gregory Beuneux:** Investigation (supporting); writing – review and editing (supporting). **Jean‐François Desmet:** Investigation (supporting); writing – review and editing (supporting). **Cécile Kaerle:** Data curation (supporting); investigation (supporting). **Guillaume Queney:** Data curation (equal); investigation (equal). **Michel Barataud:** Conceptualization (supporting); data curation (lead); writing – review and editing (supporting). **Dominique Pontier:** Conceptualization (lead); project administration (lead); writing – original draft (equal); writing – review and editing (equal).

## CONFLICT OF INTEREST

The authors declare no competing interest in this work.

## Supporting information


Appendix S1

## Data Availability

The generated *Cyt‐b* sequences have been deposited in the GenBank database (accession numbers Banklt2637337: OP763182–OP763342). Microsatellites' genotypes will be made available on the Dryad repository.
