# Supplementary material for: Species delineation and genetic structure of two Chaerephon species (C. pusillus and C. leucogaster) on Madagascar and the Comoro archipelago
Source: Ecol Evol. 2022 Dec 3;12(12):e9566. doi: 10.1002/ece3.9566 (PMC9719067; doi:10.1002/ece3.9566)
Supplement: Supplementary file 1 — Appendix S1 [file ECE3-12-e9566-s001.docx]

Supplementary Information

**Species delineation and genetic structure of two *Chaerephon* species (*C. pusillus* and *C. leucogaster*) on Madagascar and the Comoro archipelago**

Morgane Tidière*, Elodie Portanier*, Stéphanie Jacquet*, Steven M. Goodman, Gildas Monnier, Grégory Beuneux, Jean-François Desmet, Cécile Kaerle, Guillaume Queney, Michel Barataud, and Dominique Pontier

**Table S1. Primers used to PCR amplify the cytochrome b and five microsatellite markers for the 161 and 200 sampled individuals of *Chaerephon* species, respectively.**

| **Microsatellite locus** | **Publication** | **Forward primer 5’ > 3’** | **Reverse primer 5’ > 3’** | **Fluorescent**  **Dye** | **Final concentration (µM)** |
| --- | --- | --- | --- | --- | --- |
| Cyt-b |  | CTGCCACAGCATTCAACTCA | GTTGGCTGGGGTGTAATTGT |  | 0.20 |
| TabrA10 | Naidoo *et al.* 2013 | AAGTGGTTGGGCGTTGTC | GTGTCTTGCGATGCACTGCCTTGAGA | 6FAM | 0.20 |
| TabrD10 | Naidoo *et al.* 2013 | CCCCACTCATTTATCCATCCACA | GTGTCTTATCTCGCAGCTATTGAAGTA | 6FAM | 0.20 |
| TabrD15 | Naidoo *et al.* 2013 | AGTCCTGGCTCCTATTCTCATTG | GTGTCTTCTATCCGTCTACCTGTCCGTCTAT | 6FAM | 0.60 |
| TabrE9 | Naidoo *et al.* 2013 | GTTTGTCTTCCCCACTGA | GTGTCTTCTTAGGACAGGAGAAGTCA | 6FAM | 0.20 |
| TabrH6 | Naidoo *et al.* 2013 | ATCTCTCCAGTCCTTACCA | GTGTCTTTTTACCCTCCACAGTCTCA | 6FAM | 0.20 |

Reference for Table S1:

Naidoo, T., Macdonald, A., & Lamb, J.M. (2013). Cross-genus amplification and characterisation of microsatellite loci in the little free tailed bat, *Chaerephon pumilus* s. l. (Molossidae) from South Eastern Africa. *African Journal of Biotechnology, 12*, 3143-3147

**Table S2. Prior distributions of demographic, historical and mutation parameters used for ABC inferences.**

| **Parameter description** | **Parameter** | **Prior distribution** |
| --- | --- | --- |
| Stable effective population size | Npum, Npus, Nl | Loguniform [1000; 200,000] |
| Duration of bottleneck | Db | Uniform [1; 2000] |
| Recent time of migration | Tmr | Uniform [500; 25,000] |
| Ancient time of migration | Tma | Uniform [200,000; 800,000] |
| Divergence time | TdL, TdP | Uniform [200,000; 2,500,000] |
| Microsatellite mutation rate | µm | Loguniform [1.10^-5^; 10^-3^] |
| Mitochondrial mutation rate | µc | Loguniform [1.10^-8^; 10^-5^] |
| Migration rate | m | Loguniform [10^-6^; 10^-1^] |

Effective population sizes (N) are expressed in number of diploid individuals and times of events (t) in number of generations going back to the past. Conditions among the parameters used during the simulations were TdL and TdP > Tma and Tmr.

**Table S3. Null alleles frequencies for the five microsatellite markers for *Chaerephon* species**. Estimations were made using the 136 *C. pusillus* and the 56 *C. leucogaster* morphologically identified without ambiguity. An excess of homozygotes was detected at locus 1 in both species and at locus 5 for *C. leucogaster* only.

| **Locus** | **Oosterhout** |
| --- | --- |
| *C. pusillus* (n=136) |  |
| TabrA10 | 0.1034 |
| TabrD10 | -0.0164 |
| TabrD15 | 0.0318 |
| TabrE9 | -0.0185 |
| TabrH6 | -0.0222 |
| *C. leucogaster* (n=56) |  |
| TabrA10 | 0.1056 |
| TabrD10 | -0.0162 |
| TabrD15 | 0.0296 |
| TabrE9 | -0.0343 |
| TabrH6 | 0.0081 |

**Table S4. Pairwise *Fst* values for *Chaerephon leucogaster* (n=56) sampled in three different regions of Madagascar (former provincial names given) that were formerly administered at the provincial level.** All pairwise *Fst* values were significantly different from zero.

|  | Mahajanga | Toliara |
| --- | --- | --- |
| Antsiranana | **0.0420** | **0.0421** |
| Mahajanga | 0 | **0.0258** |

**Table S5. Pairwise Fst values for *Chaerephon pusillus* (n=136) sample on the four islands in the Comoro archipelago.** All pairwise Fst values were significantly different from zero.

|  | Grande Comore | Mohéli | Mayotte |
| --- | --- | --- | --- |
| Anjouan | **0.0738** | **0.1043** | **0.1183** |
| Grande Comore | 0.0000 | **0.0460** | **0.0914** |
| Mohéli |  | 0.0000 | **0.0891** |

**Figure S1. Variability of the size of the white patch in individuals captured in Mayotte.**


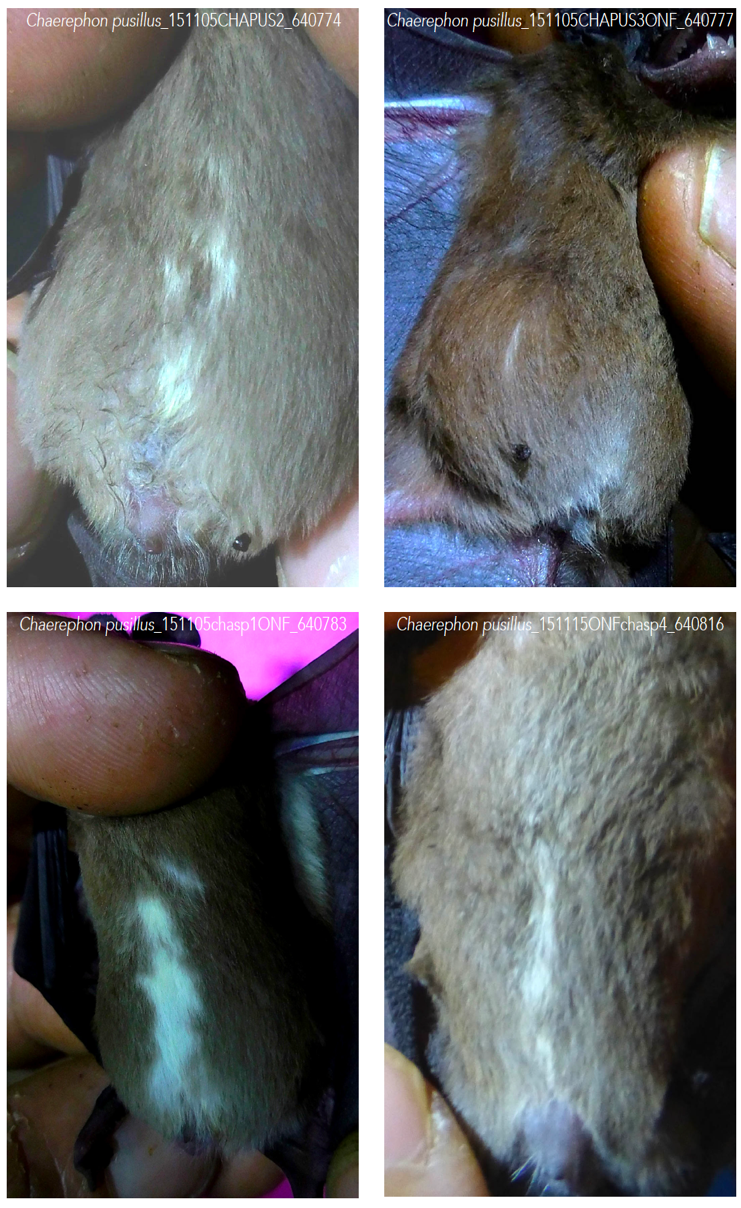

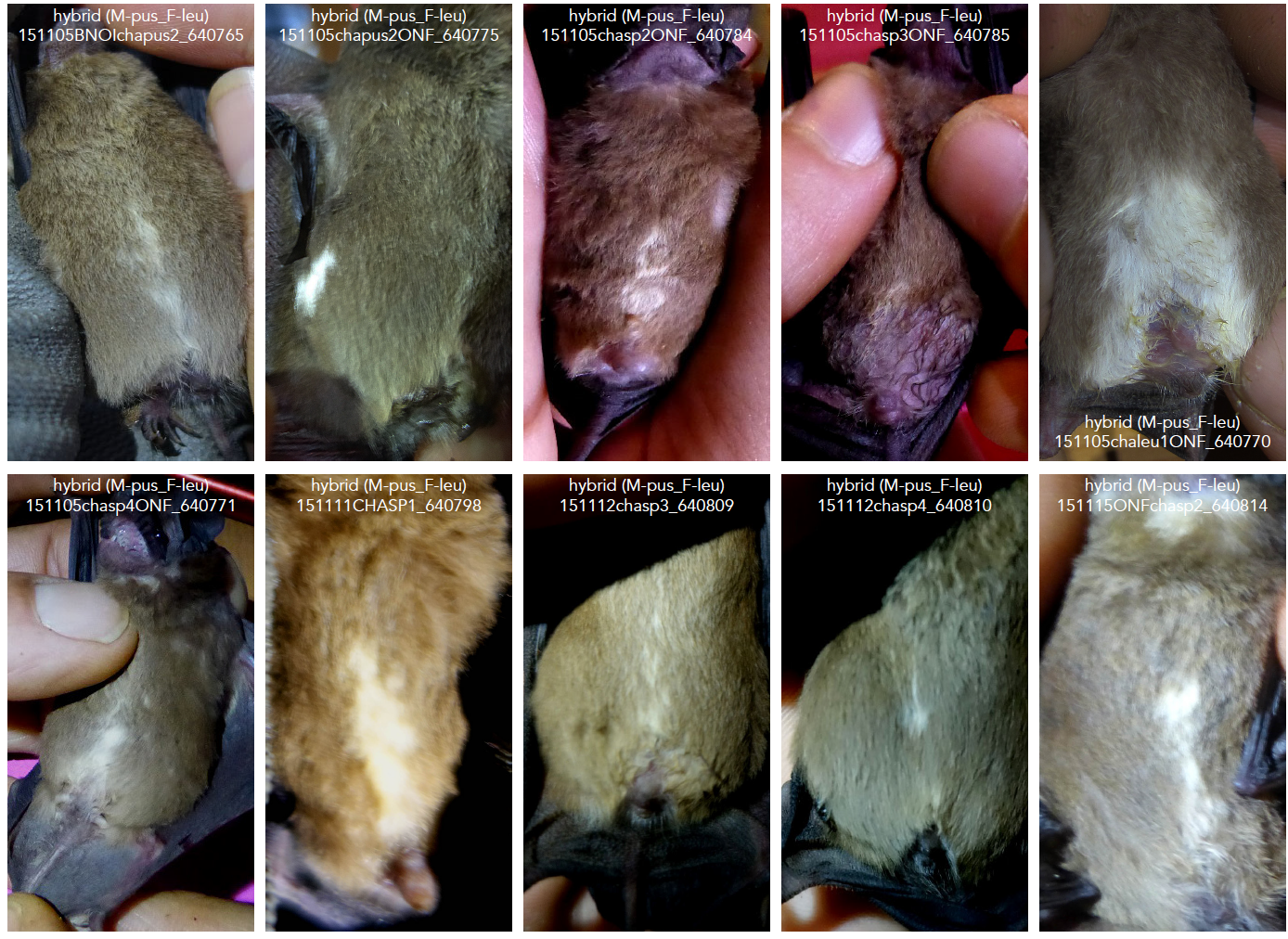


**Figure S2. Phylogenetic tree of *Chaerephon* species based on cytochrome b sequences of 234 individuals, including 161 sequences generated in this study.** Node values correspond to bootstrap analyses. Nodes supported by more than 70% are indicated. Individuals sequenced in this study are highlighted in blue, in red and in purple for *C. pusillus*, *C. leucogaster* and *C. pusillus* – *leucogaster* hybrids, respectively. The remaining sequences in black are publicly available sequences from both species, and from *C. pumilus*.

**
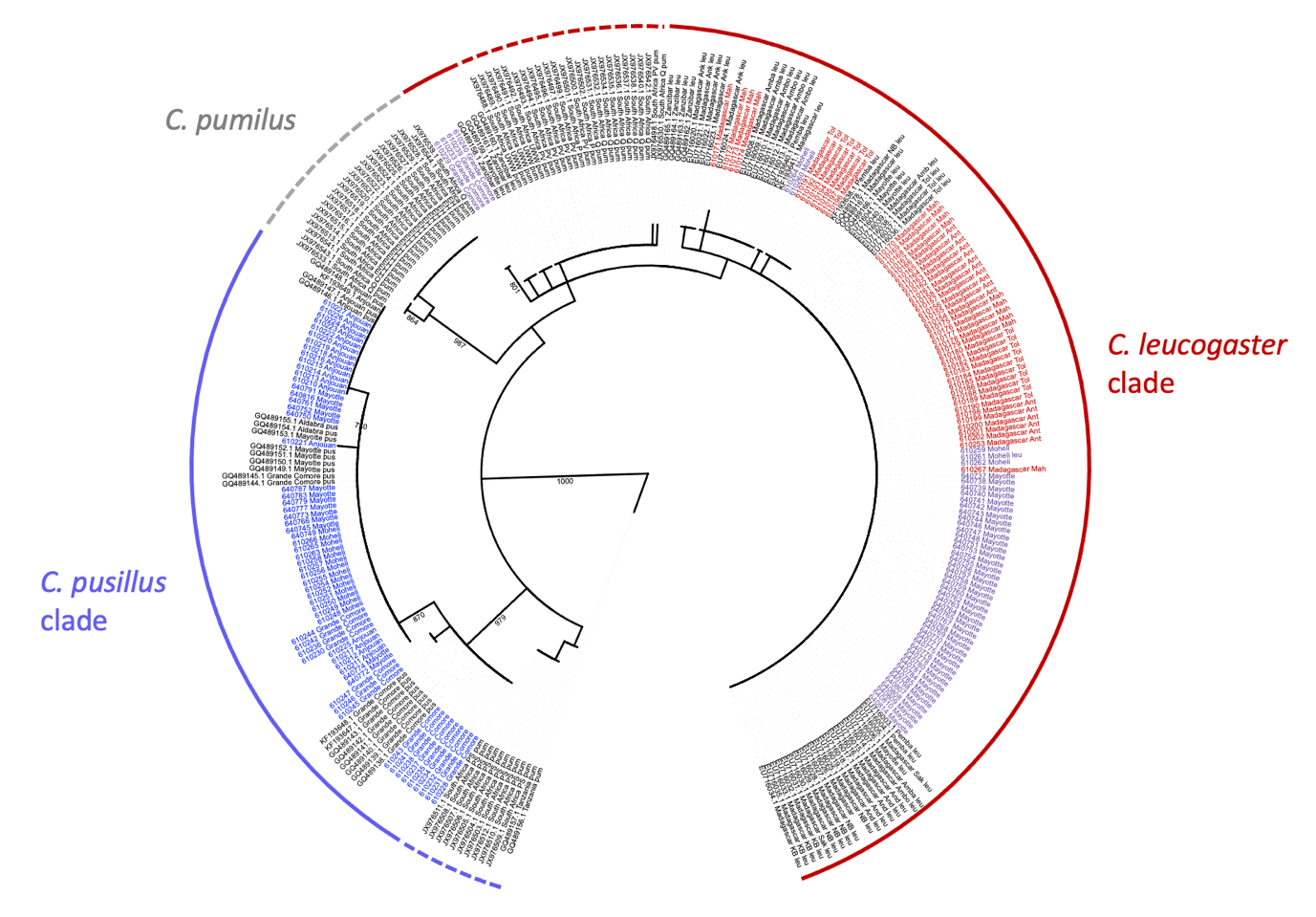
**

**Figure S3. Likelihood (A) and delta K (B) for each *K* tested using STRUCTURE software for the 200 individuals of *Chaerephon* species.** The maximum likelihood method pointed *K*=3 as optimal clustering solution but *K*=2 received similar support, while the Evanno method indicated two clusters.


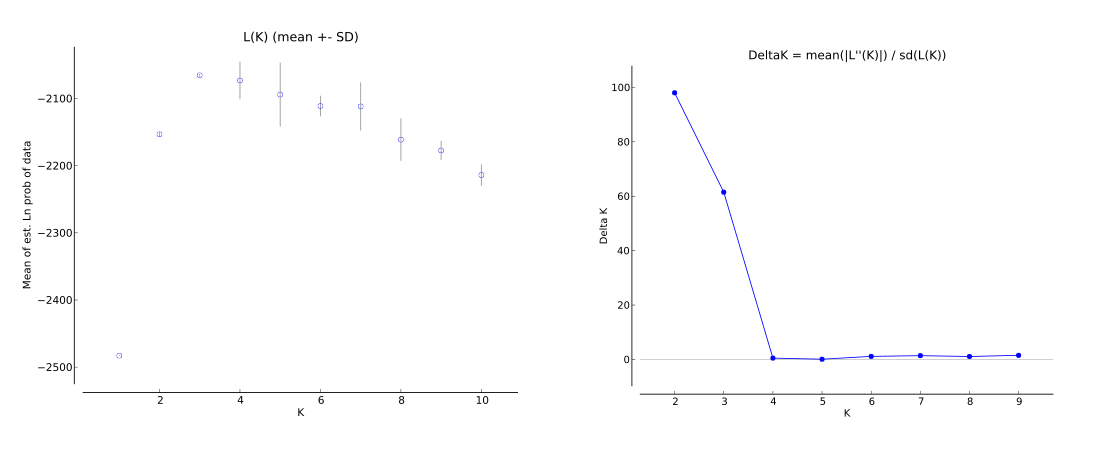

**Figure S4.** **PCA goodness of fit for the best fitting model**. The graphic represents the 90% envelope of the PCA with two components. The black cross represents the real data set, and the circle lines represent the simulation dataset.

**Figure S5.** **Confusion matrix indicating the percentage of misclassification of the models.** In brackets is the probability of selecting the correct model for each scenario. The same result was obtained with both data sets.


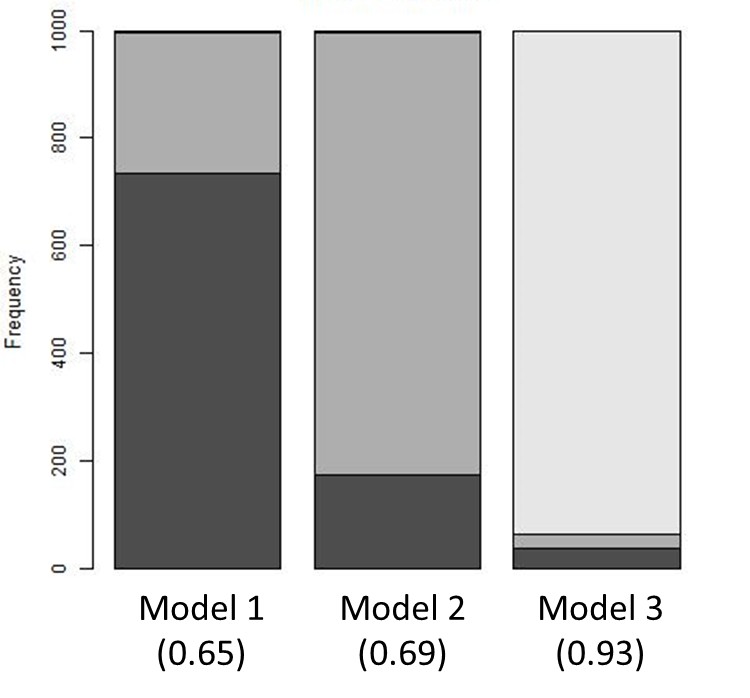


**Figure S6. Bayesian skyline plot (BSP) based on *Cyt-b* in *Chaerephon leucogaster* (a) and *C. pusillus* (b)**. The x-axis indicates times in years before the present, and the y-axis shows the effective population size. The black line represents the median population size, and the blue range indicates 95% posterior intervals for the population size change.


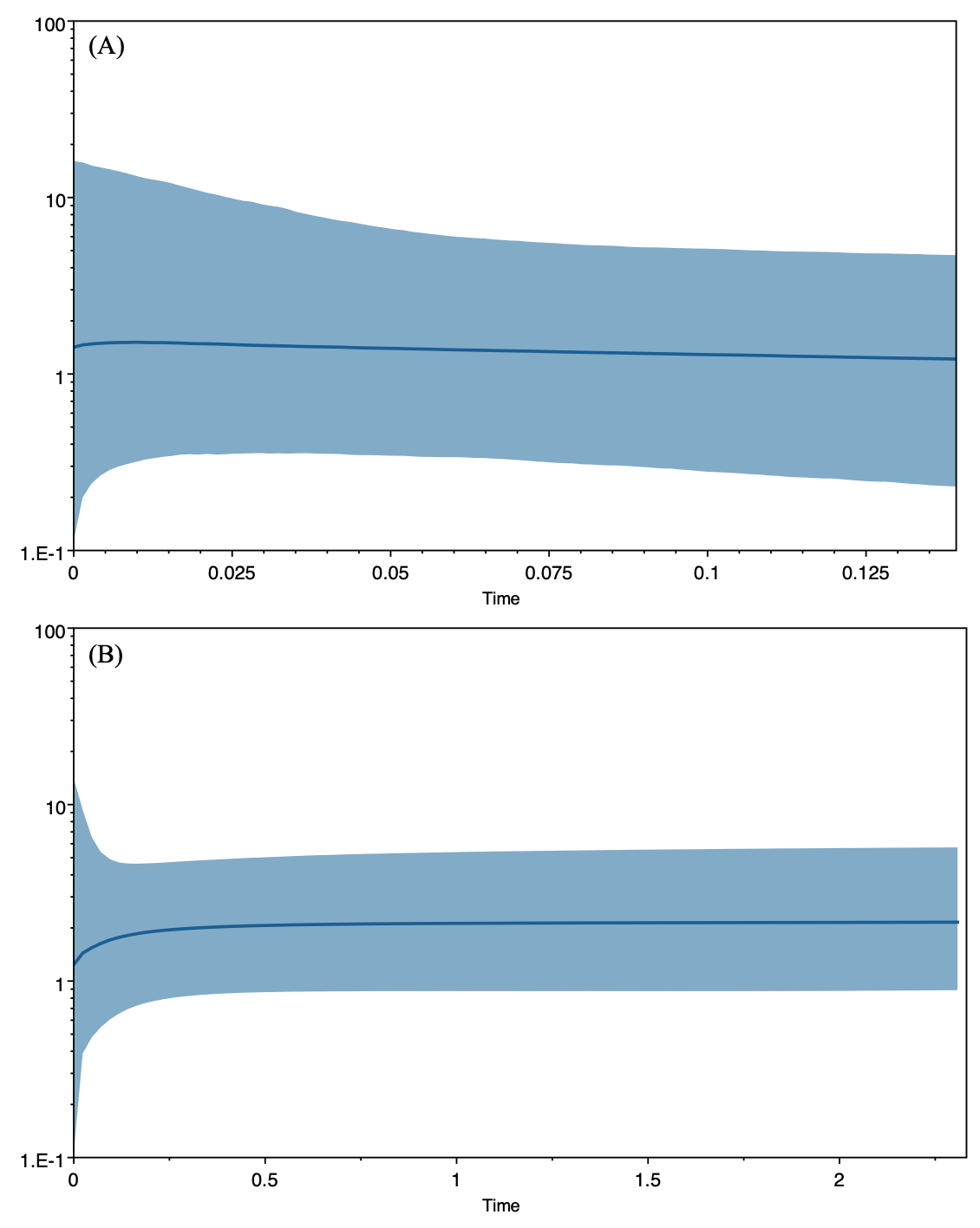


**Figure S7. Allelic richness according to the island or district for individuals assigned to *Chaerephon pusillus* and *C. leucogaster* based on STRUCTURE method.** Allelic richness tended to be lower in *C. pusillus* than in *C. leucogaster*.


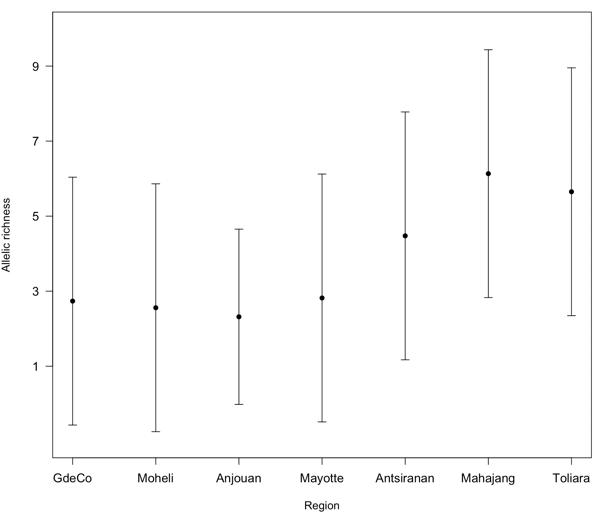


***C. leucogaster***

***C. pusillus***


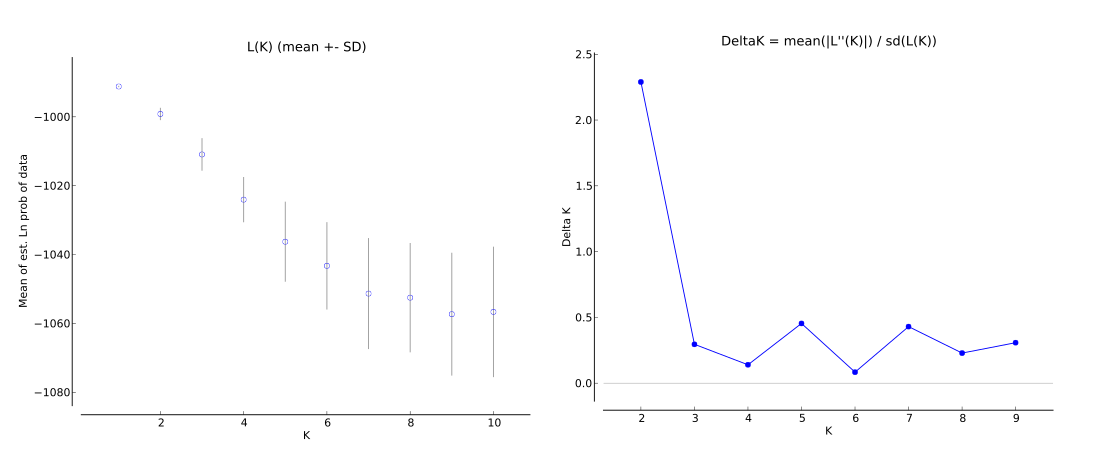
**Figure S8. Likelihood and delta K for each *K* were tested using STRUCTURE software for the 56 individuals of *Chaerephon leucogaster* sampled on Madagascar.** The maximum likelihood method indicated only one cluster, while Evanno’s method indicated two clusters.


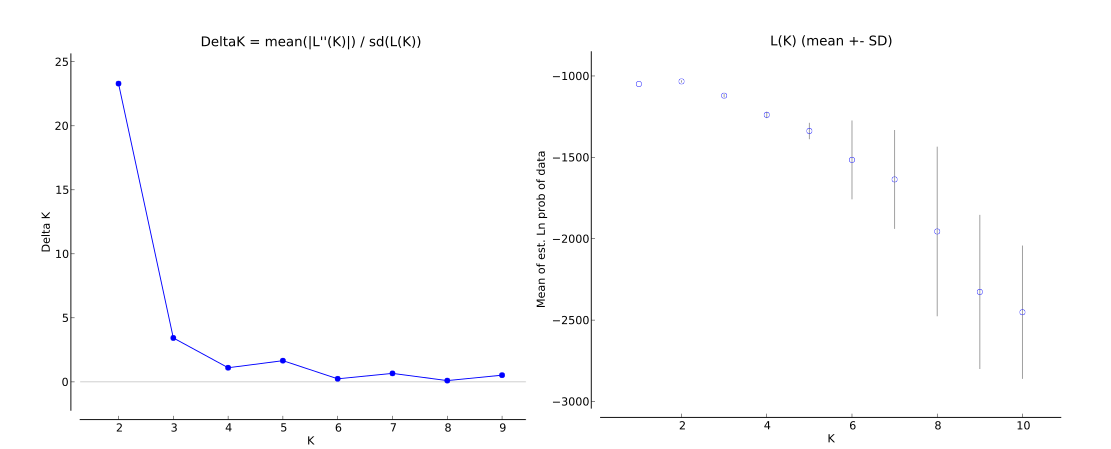
**Figure S9. Likelihood and delta K for each *K* tested using STRUCTURE software for the 144 individuals of *Chaerephon pusillus* sampled in the Comoro archipelago.** The maximum likelihood method indicated only one cluster, while the Evanno’s method indicated three clusters.

**Figure S10. Membership probabilities for the 56 individuals sampled on Madagascar of *Chaerephon leucogaster* species in two clusters.** No genetic structure was identified.


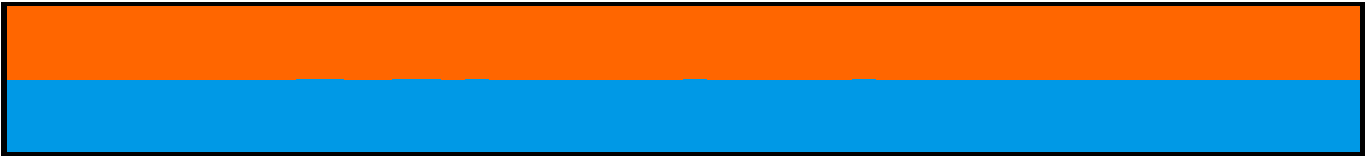


**Figure S11. Assignment probabilities to three clusters for the 144 individuals of *Chaerephon pusillus* captured in the Comoro archipelago.** No genetic structure was identified.


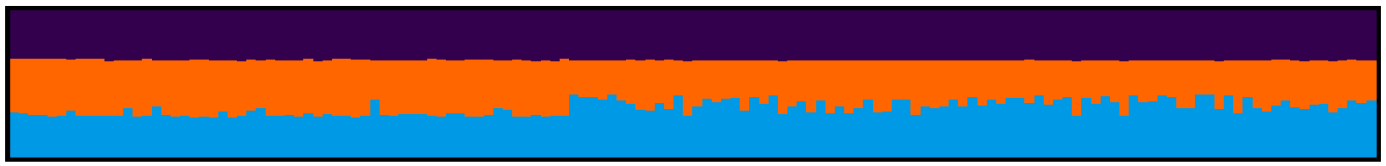


**Figure S12. Principal Component Analysis based on the (A) morphological identification, (B) *Cyt-b* assignation, and (C) presence or absence of white fur patch in 82 individuals captured on Mayotte.** The three morphological measures considered to perform the PCA are the length of the forearm (AD), the third finger (D3) and the fifth finger (D5). No distinction between individuals with or without the white fur patches was demonstrated based on these three morphological criteria at least.


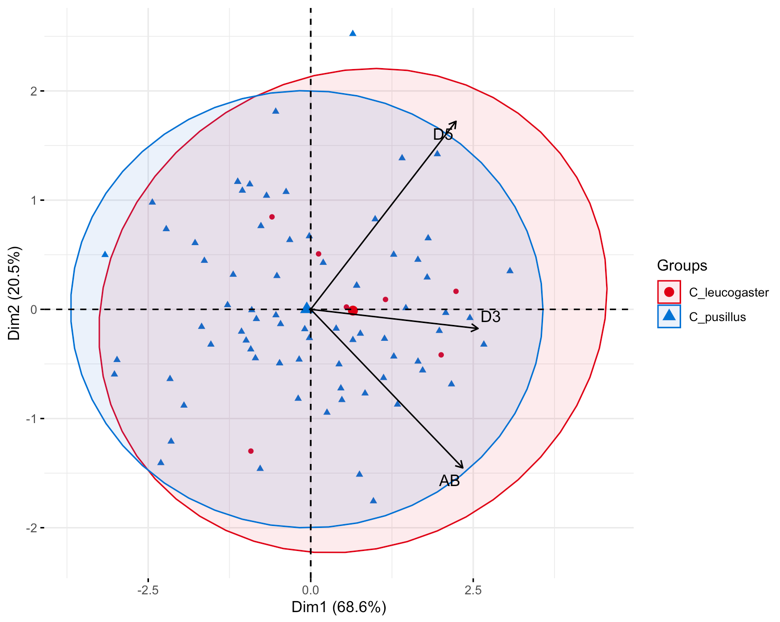

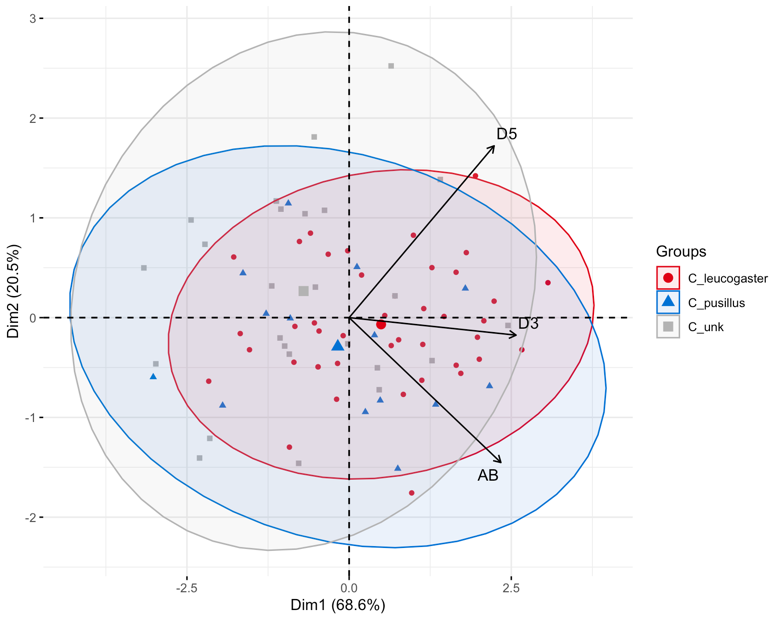

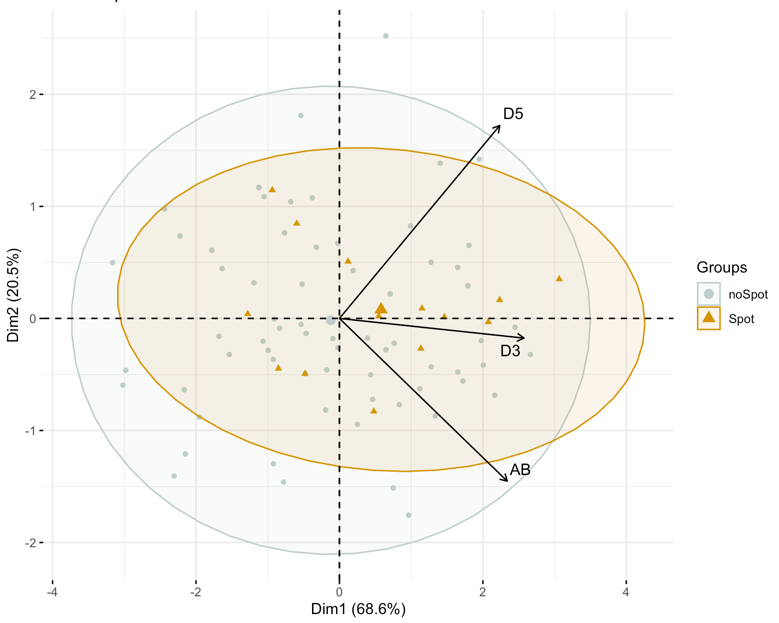


(A)

(B)

(C)
